# Genome-wide association study of circulating levels of glucagon during an oral glucose tolerance test

**DOI:** 10.1186/s12920-020-00841-7

**Published:** 2021-01-06

**Authors:** Anna Jonsson, Sara E. Stinson, Signe S. Torekov, Tine D. Clausen, Kristine Færch, Louise Kelstrup, Niels Grarup, Elisabeth R. Mathiesen, Peter Damm, Daniel R. Witte, Marit E. Jørgensen, Oluf Pedersen, Jens Juul Holst, Torben Hansen

**Affiliations:** 1 The Novo Nordisk Foundation Center for Basic Metabolic Research, University of Copenhagen, Blegdamsvej 3B, 2200 Copenhagen, Denmark; 2 Department of Biomedical Sciences, Faculty of Health and Medical Sciences, University of Copenhagen, Copenhagen, Denmark; 3 Department of Gynecology and Obstetrics, Nordsjaellands Hospital, University of Copenhagen, 3400 Hilleroed, Denmark; 4 Department of Clinical Medicine, Faculty of Health and Medical Sciences, University of Copenhagen, 2200 Copenhagen, Denmark; 5 Steno Diabetes Center Copenhagen, Gentofte, Denmark; 6 Department of Obstetrics, Copenhagen University Hospital, Rigshospitalet, Copenhagen, Denmark; 7 Center for Pregnant Women with Diabetes, Copenhagen University Hospital, Rigshospitalet, Copenhagen, Denmark; 8 Department of Endocrinology, Copenhagen University Hospital, Rigshospitalet, Copenhagen, Denmark; 9 The Danish Diabetes Academy, Odense, Denmark; 10 Institute of Public Health, University of Aarhus, Aarhus, Denmark; 11 Steno Diabetes Center Aarhus, Aarhus, Denmark; 12 National Institute of Public Health, University of Southern Denmark, Odense, Denmark

**Keywords:** Glucagon, GWAS, Type 2 diabetes

## Abstract

**Background:**

In order to explore the pathophysiology underlying type 2 diabetes we examined the impact of gene variants associated with type 2 diabetes on circulating levels of glucagon during an oral glucose tolerance test (OGTT). Furthermore, we performed a genome-wide association study (GWAS) aiming to identify novel genomic loci affecting plasma glucagon levels.

**Methods:**

Plasma levels of glucagon were examined in samples obtained at three time points during an OGTT; 0, 30 and 120 min, in two separate cohorts with a total of up to 1899 individuals. Cross-sectional analyses were performed separately in the two cohorts and the results were combined in a meta-analysis.

**Results:**

A known type 2 diabetes variant in *EYA2* was significantly associated with higher plasma glucagon level at 30 min during the OGTT (Beta 0.145, SE 0.038, *P* = 1.2 × 10^–4^) corresponding to a 7.4% increase in plasma glucagon level per effect allele. In the GWAS, we identified a marker in the *MARCH1* locus, which was genome-wide significantly associated with reduced suppression of glucagon during the first 30 min of the OGTT (Beta − 0.210, SE 0.037, *P* = 1.9 × 10^–8^), equivalent to 8.2% less suppression per effect allele. Nine additional independent markers, not previously associated with type 2 diabetes, showed suggestive associations with reduced glucagon suppression during the first 30 min of the OGTT (*P* < 1.0 × 10^–5^).

**Conclusions:**

A type 2 diabetes risk variant in the *EYA2* locus was associated with higher plasma glucagon levels at 30 min. Ten additional variants were suggestively associated with reduced glucagon suppression without conferring increased type 2 diabetes risk.

## Background

Glucagon is a key regulator of hepatic glucose production and is therefore intimately linked to type 2 diabetes pathophysiology. Key regulators of glucagon secretion are insulin and glucose [1]. Patients with type 2 diabetes have disproportionately elevated fasting glucagon levels and also exhibit reduced early glucose-induced suppression of glucagon secretion in response to an oral glucose tolerance test (OGTT) [2]. These alterations in glucagon secretion have been proposed to represent a primary defect but may be secondary to other metabolic defects in type 2 diabetes [2, 3].

Genetic influence on glucagon secretion in response to a glucose challenge has not been widely studied in large populations, mostly due to the strict sample handling protocol necessary to preserve the labile hormone and obtain valid results along with the use of validated assays. The most comprehensive genome-wide association study (GWAS) for glucagon so far was reported by Almgren et al. [4] in 2017. They performed a meta-analysis on circulating levels of glucagon measured at two time points, 0 and 120 min, during an OGTT including 3344 Swedish individuals from Malmö Diet and Cancer study (MDC) and 4905 Finnish individuals from the Prevalence, Prediction and Prevention of diabetes Botnia study (PPP-Botnia). Six suggestively associated loci to glucagon levels were reported, four for fasting levels of glucagon and two for 2 h glucagon levels [4].

Here we investigated the impact of genetic variants on circulating plasma levels of glucagon at three time points, 0, 30 and 120 min, during an OGTT. In order to explore the pathophysiology underlying type 2 diabetes, we examined the impact of known gene variants demonstrated to associate with type 2 diabetes. In addition, we tested three suggestive loci for fasting and 2 h plasma glucagon levels reported previously. Furthermore, we performed a GWAS aiming to identify novel genomic loci affecting glucagon plasma levels.

## Methods

### Study population

The ADDITION-PRO study (2009–2011) [5] is a continuation of the Danish arm of the ADDITION study (2001–2006) [6], in which individuals with normal glucose tolerance (NGT), impaired fasting glycaemia (IFG), impaired glucose tolerance (IGT) or type 2 diabetes (free from medical treatment) are followed. In total, 2082 participants have undergone an extensive examination, including detailed characterization of glycemic status based on a 3-point OGTT at baseline. Information regarding both glucagon levels and GWAS was available in 1346 individuals. Absolute glucagon levels and the percentage of glucagon suppression during the OGTT in individuals with different glycemic status have been reported previously [7]. Ethical approval was obtained from the Scientific Ethics Committee of the Central Denmark Region (journal no. 20080229), and all participants provided oral and written informed consent before participating in the study.

The Rigshospitalet cohort (RigCoh) [8] is a cohort of 597 adult offspring to mothers with either gestational diabetes, type 1 diabetes or no diabetes during pregnancy. All participants were born at the Department of Obstetrics, Rigshospitalet, Denmark, from 1978 to 1985. Only singletons above the age of 18 years were included, and if more than one sibling from the study period met criteria for inclusion, only the oldest was invited. After an overnight fast, participants without diagnosed diabetes had a standard 75 g OGTT, with venous plasma samples collected at 0, 30 and 120 min. Measurements of plasma glucagon were available from 567 participants of the study [9]. In total, 553 individuals with available data on glucagon levels and GWAS data were included in the analysis. The protocol was in accordance with the Declaration of Helsinki and approved by the local ethical committee (journal no. KF01-061/03). All participants gave a written consent before taking part in the study. Clinical characteristics of the study participants are provided in Table 1.

**Table 1:** Clinical characteristics of the study cohorts

	ADDITION-PRO	RigCoh
N (M/F)	1346 (709/637)	553 (264/289)
Age (years)	66.3 (6.9)	22.0 (2.2)
BMI (kg/m^2^)	27.2 (4.6)	24.5 (4.8)
Fasting plasma glucose (mmol/l)	5.9 [1.0]	5.3 [0.6]
HbA1c (%)	5.7 [0.4]	5.0 [0.3]
HbA1c (mmol/mol)	38.8 [4.4]	31.1 [3.3]
Fasting plasma glucagon (pmol/l)	10.0 [7.0]	6.5 [4.0]
30 min plasma glucagon (pmol/l)	8.0 [6.0]	5.0 [4.0]
2 h plasma glucagon (pmol/l)	6.0 [4.0]	3.0 [3.0]

Data are mean ± standard deviation or median [inter quartile range]

### Measurements

Height and weight were measured as previously described [5] and BMI calculated as weight in kilograms divided by height in meters squared. Blood samples were drawn at 0, 30 and 120 min during a standard 75 g OGTT after an overnight fast of ≥ 8 h for assessment of serum insulin and plasma glucose and glucagon concentrations. Blood samples for measurement of glucagon were drawn into EDTA tubes and were put on ice immediately and frozen. Radioimmunological determination of total plasma glucagon concentration was performed as previously described [10] using an antibody against the C-terminus of glucagon and therefore devoid of cross-reactivity with gut derived proglucagon moieties (glicentin and oxyntomodulin). The analytical detection limit was 1 pmol/l, sensitivity around 1 pmol/l and intra- and inter-assay coefficients of variation were < 6% and  < 15%, respectively. The applied methodology was recently evaluated [11]. Glycated haemoglobin A1c (HbA1c) was measured by high-performance liquid chromatography (HPLC) as described previously [5].

The decremental areas under the curves (dAUCs) of plasma glucagon concentrations were calculated using the trapezoid rule, from 0 to 30 min (dAUC 0–30 min) and 0 to 120 min (dAUC 0–120 min) during the OGTT. In a secondary analysis, we calculated the fractional change in plasma glucagon levels from 0 to 30 and from 0 to 120 min by dividing the value at 30 min with that at fasting.

### Genotyping

A total of 1899 individuals from the two cohorts were genotyped by the Illumina Infinium HumanCoreExome Beadchip platform (Illumina, San Diego, CA). Genotypes were called using the Genotyping module (version 1.9.4) of GenomeStudio software (version 2011.1, Illumina). Closely related individuals, individuals with an extreme inbreeding coefficients, individuals with mislabeled sex, individuals with a call rate < 95%, duplicates and individuals identified as ethnic outliers, as well as genetic markers with a call rate < 98%, a minor allele frequency < 0.01 and a Hardy–Weinberg equilibrium *P* < 1 × 10^–5^, were removed during quality control. Imputation was performed on the Michigan imputation server [12] using haplotypes from the Haplotype Reference Consortium (HRC version r1.1) panel [13].

### Statistical analysis

Associations between genetic variants and plasma levels of glucagon were studied using a linear mixed model (EMMAX) implemented in the EPACTS software package [14] by the use of inverse-normalized residuals of trait values adjusted for age, sex and BMI in each cohort separately. Individuals with missing information were excluded from the analysis. The results were subsequently combined in a meta-analysis using METAL (http://csg.sph.umich.edu/abecasis/Metal). *P* values below 5.0 × 10^–8^ were considered genome-wide significant, while *P* values < 1 × 10^–5^ were considered to be suggestively associated. Since the ADDITION-PRO cohort contains individuals with different glucose tolerance status, we performed the analyses with and without adjustment for glucose tolerance status as well as in individuals with normal glucose tolerance separately. The results of the analyses were similar and adjustment for glucose tolerance did not affect the results. We here present the results with adjustments for age, sex and BMI in both cohorts. We selected 403 markers that have previously been associated with type 2 diabetes [15]. We used a threshold for minor allele frequency (MAF) of < 0.01, which resulted in 367 markers left for analysis. Bonferroni adjustment was used to correct for multiple testing, while analyzing the selected variants, thus *P* < 1.4 × 10^–4^ was considered to be significant in those analyses. For the replication analyses of genetic variants previously reported to be suggestively associated with plasma glucagon levels, *P* values < 0.05 was considered to be nominally significant. The presented *P* values are uncorrected.

## Results

### Known type 2 diabetes associated variant associates with increased 30 min glucagon levels

When analyzing the variants previously reported to associate with type 2 diabetes in the paper by Mahajan et al. [15], the variant in the *EYA2* locus was associated with higher 30 min plasma glucagon levels during the OGTT, after correction for multiple testing (rs6063048 Beta 0.145, SE 0.038, *P* = 1.2 × 10^–4^, see Additional file 1: Table S1). None of the selected variants were significantly associated with either fasting or 120 min glucagon levels.

### Nominal association of previously reported glucagon associated variant

We identified a nominal association for the variant rs28929474 on chromosome 14 in *SERPINA1*, reported by Almgren et al. [4] to be associated with increased fasting glucagon levels. In our meta-analysis, we could replicate the reported association of the T-allele of rs28929474 with increased fasting glucagon levels (Beta 0.345, SE 0.114, *P* = 0.0024, see Additional file 1: Table S2). Three of the remaining suggestive variants for fasting and 120 min glucagon levels were excluded in our data set due to low allele frequency. Results for the remaining three markers can be found in Additional file 1: Table S2.

### Novel gene locus associated with reduced suppression of early glucagon secretion

The strongest association was found for early glucagon suppression (dAUC 0–30 min). The C-allele of rs4691991 in the *MARCH1* locus was genome-wide significantly associated with reduced suppression of glucagon during the first 30 min of the OGTT (Beta − 0.210, SE 0.037, *P* = 1.9 × 10^–8^, see Table 2) corresponding to 8.2% reduction per effect allele. We also discovered a suggestive locus for dAUC 0–30 min of glucagon on chromosome 7, between *PRKAG2* and *GALNTL5* (Beta -0.334, SE 0.067, *P* = 6.9 × 10^–7^). Other suggestive loci for dAUC 0–30 min of glucagon can be found in Table 2. The suggestive associations for fasting, 30 and 120 min glucagon as well as dAUC 0–120 min for glucagon are shown in Additional file 1: Table S3–6.

**Table 2:** Genome-wide significant and suggestive loci for dAUC 0–30 min of plasma glucagon levels during an OGTT

Chr	Position	SNP	Closest gene	Distance	EA	NEA	EAF	Beta	SE	*P*-value
*Genome-wide significant association*										
4	165473425	rs4691991	*MARCH1*	169 kb	C	G	0.29	− 0.210	0.037	1.90 × 10^–8^
*Suggestive associations*										
7	151616838	rs7794104	*PRKAG2/GALNTL5*	42 kb/36 kb	T	C	0.92	− 0.334	0.067	6.89 × 10^–7^
9	98523829	rs7864076	*ERCC6L2*	114 kb	T	C	0.49	− 0.162	0.033	9.75 × 10^–7^
18	62985882	rs17689596	*CDH7*	431 kb	T	G	0.067	− 0.316	0.065	1.11 × 10^–6^
1	181531664	rs80231151	*CACNA1E*	Intergenic	G	A	0.082	− 0.305	0.065	2.38 × 10^–6^
5	161945575	rs56820633	*GABRG2*	363 kb	A	T	0.096	0.270	0.058	2.80 × 10^–6^
13	97508453	rs16953620	*HS6ST3*	16 kb	A	G	0.86	− 0.218	0.047	3.23 × 10^–6^
15	39161381	rs12442819	*RASGRP1*	304 kb	T	G	0.29	− 0.171	0.038	7.68 × 10^–6^
6	99283376	rs195860	*POU3F2*	Intergenic	T	G	0.13	− 0.215	0.048	8.72 × 10^–6^
14	65833499	rs10149004	*FUT8*	43 kb	C	T	0.13	− 0.211	0.048	9.11 × 10^–6^

Beta (SE) from linear mixed model denotes the effect of each of the effect alleles (EA, additive model) on the inverse-normalized residuals of trait adjusted for age, sex and BMI. Effect denotes the change in percent in plasma glucagon concentration for each of the EAs obtained from a linear mixed model on the logarithmically transformed plasma levels adjusted for age, sex and BMI. *Chr* chromosome, *NEA* non effect allele, *EAF* effect allele frequency. Distance indicates how far from the gene the marker is located

## Discussion

Here we performed a GWAS of plasma levels of glucagon during an OGTT in 1899 individuals and identified a previously described type 2 diabetes variant in *EYA2,* we replicated a previously reported suggestive association with fasting glucagon level and identified a novel genome-wide significant variant, rs4691991 in the *MARCH1* locus*.*

Our results indicate that genetic influence of fasting glucagon levels and suppression of glucagon secretion during an OGTT have a limited impact on type 2 diabetes development. We only identified one of the current known type 2 diabetes risk alleles to associate with glucagon levels during OGTT. The variant in *EYA2* was associated with increased glucagon levels at 30 min during the OGTT. *EYA2* is involved in DNA repair and has, besides type 2 diabetes, also been reported to associate with triglyceride levels [16] and waist-hip ratio [17].

We plotted all the selected markers in a QQ-plot, to investigate if we had more significant associations of the type 2 diabetes risk increasing alleles to plasma glucagon levels during the OGTT than expected by chance (Additional file 2: Figure S1) and could conclude that it is most probably not the same genetic markers that is associated with type 2 diabetes that also affect glucagon levels.

We were able to replicate one of the suggestive associations with fasting glucagon level reported by Almgren et al. [4]. Importantly, in both this and the present study, the same glucagon assay has been used. One of the variants, rs142179968 in *MACF1*, for which Almgren et al. [4] found a suggestive association with fasting glucagon, has a MAF of < 0.0005 according to the UCSC Genome Browser (GRCh37/hg19) [18, 19], although it was slightly higher in the meta-analysis of the MDC and PPP-Botnia cohorts described in the paper (MAF = 0.002) [4]. Since we restricted our analyses to include only markers with a MAF > 0.01, this variant was not analyzed in our primary data set. Another variant associated with fasting glucagon in the aforementioned paper, rs140436257, located in *TDC*, has a reported allele frequency < 0.003, but is ten times more common in the meta-analysis by Almgren et al. (MAF = 0.026). However, this variant was not included in the analysis reported here and neither was the variant rs111485612 with a MAF below 1%.

We identified a genome-wide significant association with 0–30 min glucagon suppression. The closest gene to the signal is *MARCH1*, which mediates ubiquitination of proteins and promotes their subsequent endocytosis and sorting to lysosomes. Variants in *MARCH1* have previously been shown to be nominally associated with BMI and increased risk of type 2 diabetes in the UK Biobank [20]. However, whether this gene or another gene in the locus is responsible for the effect on plasma levels of glucagon remains to be discerned.

We did not find any strong evidence of an impact of previously published type 2 diabetes genetic variants on glucagon levels during OGTT, except for *EYA2*. Neither did we find a large number of novel genetic variants affecting glucagon levels during OGTT in the GWAS performed here. We have not been able to identify any previous studies investigating the heritability of glucagon levels in the fasting state and during glucose stimulation in other cohorts. The sample size in this study is modest and might be a contributing factor to the lack of strong genetic signals. Larger studies are required to get a more comprehensive picture of the genetic influence on glucagon levels during OGTT. We are also strongly encouraging heritability studies of glucagon levels in family or twin studies in the future, since there is an obvious lack of these kinds of studies published.

A larger sample size would allow for studies of polygenic risk scores (PRS), which might be clinically useful in the future. The PRS could be used to identify individuals genetically prone to deviating glucagon levels and thereby monitor them before any adverse effect manifests.

The accuracy of measurements of plasma glucagon has been debated for many years. Apart from the usual reliability concerns, specificity and sensitivity have been the main issue. Glucagon assays may react with the glucagon-containing products from the gut, glicentin and oxyntomodulin, and with proglucagon 1–61 from the pancreas as well as the gut. Regarding glucagon secretion, inhibition of secretion, e.g. by glucose, is as important as stimulation with e.g. arginine, which puts great demands on the sensitivity of assay systems. In addition, interfering matrix effects have been a problem. The assay used in this study is a radioimmunoassay directed against the C-terminus based on a C-terminal-wrapping antibody, 4305, which grants the assay complete specificity towards molecular forms with a free glucagon C-terminal which characterizes pancreatic glucagon as opposed to the gut derived peptides, as well as a sufficient sensitivity (< 1 pmol/l) to pick up all relevant changes. The assay has been validated in several recent studies against mass spectrometry [21] as well as against sandwich ELISA [22, 23]. However, in patients with major surgical operations on the human GI tract, such as gastrectomy and bypass, the sandwich ELISA has in some cases shown aberrant behavior. The radioimmunoassay (4305) also measures proglucagon 1–61 because of the identical C-terminus, but this component plays a very minor role under normal circumstances and has glucagon-like bioactivity, if any [24]. We are therefore convinced that we have used the ideal assay for measurement of glucagon in this study [11].

A strength of our study is the measurement of the physiological important 30 min glucagon levels during an OGTT. In fact, the strongest genetic influence in our data set, was seen for plasma levels of glucagon at 30 min of the OGTT. However, it is a limitation to the study that we have not been able to validate our suggestive associations at this time point in additional cohorts, since plasma glucagon at 30 min is a rare measurement.

## Conclusions

The present meta-analysis identified a genome-wide significant association to suppression of early glucagon secretion during OGTT for a locus containing *MARCH1*. Type 2 diabetes risk variant in the *EYA2* locus associated with plasma glucagon levels at 30 min of the OGTT, while other variants influence glucagon levels without conferring increased type 2 diabetes risk. More studies of the genetics underlying fasting glucagon levels and levels of glucagon during an OGTT are needed in order to understand the genetic regulation of α-cell function and to develop genetic tools for mendelian randomization to examine the causal effect of glucagon in various metabolic pathways.

## Supplementary Information

**Additional file 1: Table S1**. Title of data: Impact of gene variants associated with type 2 diabetes on circulating levels of glucagon during an oral glucose tolerance test (OGTT). Description of data: Beta (SE) from linear mixed model denotes the effect of each of the effect alleles (EA, additive model) on the inverse-normalized residuals of trait adjusted for age, sex and BMI. NEA - non effect allele, CHR - chromosome, POS - position, EAF - effect allele frequency. **Table S2.** Title of data: Replication of previously reported markers associating with plasma glucagon levels during an oral glucose tolerance test (OGTT). Description of data: Beta (SE) from linear mixed model denotes the effect of each of the effect alleles (EA, additive model) on the inverse-normalized residuals of trait adjusted for age, sex and BMI. NEA - non effect allele, CHR - chromosome, POS - position, EAF - effect allele frequency. **Table S3**. Title of data: Suggestive loci for fasting plasma glucagon levels. Description of data: Beta (SE) from linear mixed model denotes the effect of each of the effect alleles (EA, additive model) on the inverse-normalized residuals of trait adjusted for age, sex and BMI. NEA - non effect allele, CHR - chromosome, POS - position, EAF - effect allele frequency. **Table S4**. Title of data: Suggestive loci for 30 minute plasma glucagon levels during an oral glucose tolerance test (OGTT). Description of data: Beta (SE) from linear mixed model denotes the effect of each of the effect alleles (EA, additive model) on the inverse-normalized residuals of trait adjusted for age, sex and BMI. NEA - non effect allele, CHR - chromosome, POS - position, EAF - effect allele frequency. **Table S5**. Title of data: Suggestive loci for 120 minutes plasma glucagon levels during an oral glucose tolerance test (OGTT). Description of data: Beta (SE) from linear mixed model denotes the effect of each of the effect alleles (EA, additive model) on the inverse-normalized residuals of trait adjusted for age, sex and BMI. NEA - non effect allele, CHR - chromosome, POS - position, EAF - effect allele frequency. **Table S6**. Title of data: Suggestive loci for dAUC 0-120 minute of plasma glucagon levels during an oral glucose tolerance test (OGTT). Description of data: Beta (SE) from linear mixed model denotes the effect of each of the effect alleles (EA, additive model) on the inverse-normalized residuals of trait adjusted for age, sex and BMI. NEA - non effect allele, CHR - chromosome, POS - position, EAF - effect allele frequency.

**Additional file 2: Figure S1.** Title of data: QQ-plot of selected variants previously reported to associate with increased risk of type 2 diabetes. Description of data: The figure shows the observed vs. the expected P-values of the selected variants for fasting glucagon (A), 30 min glucagon (B), 120 min glucagon (C), decremental area under the curve (dAUC) 0-30 minutes of glucagon (D) and dAUC 0-120 minutes of glucagon (E) during the OGTT. There was no obvious excess of genetic variants, previously associated with type 2 diabetes, associating with circulating glucagon during OGTT.

## Data Availability

The datasets generated and analyzed during the current study are not publicly available due to data protection regulations in Denmark, but are available from the corresponding author on reasonable request.

## Abbreviations

OGTT: Oral glucose tolerance test
GWAS: Genome-wide association study
*EYA2*: EYA transcriptional coactivator and phosphatase 2
*MARCH1*: Membrane associated ring-CH-type finger 1
MDC: Malmö Diet and Cancer study
PPP-Botnia: Prevalence, Prediction and Prevention of diabetes Botnia study
NGT: Normal glucose tolerance
IFG: Impaired fasting glycaemia
IGT: Impaired glucose tolerance
RigCoh: Rigshospitalet cohort
HbA1c: Glycated haemoglobin A1c
HPLC: High-performance liquid chromatography
dAUC: Decremental area under the curve
MAF: Minor allele frequency
EA: Effect allele
Chr: Chromosome
NEA: Non effect allele
EAF: Effect allele frequency
