# Supplementary material for: Genome-wide association study of circulating levels of glucagon during an oral glucose tolerance test
Source: BMC Med Genomics. 2021 Jan 6;14:3. doi: 10.1186/s12920-020-00841-7 (PMC7788944; doi:10.1186/s12920-020-00841-7)
Supplement: Supplementary file 2 — Additional file 2: Figure S1. Title of data: QQ-plot of selected variants previously reported to associate with increased risk of type 2 diabetes. Description of data: The figure shows the observed vs. the expected P-values of the selected variants for fasting glucagon (A), 30 min glucagon (B), 120 min glucagon (C), decremental area under the curve (dAUC) 0-30 minutes of glucagon (D) and dAUC 0-120 minutes of glucagon (E) during the OGTT. There was no obvious excess of genetic variants, previously associated with type 2 diabetes, associating with circulating glucagon during OGTT. [file 12920_2020_841_MOESM2_ESM.pdf]

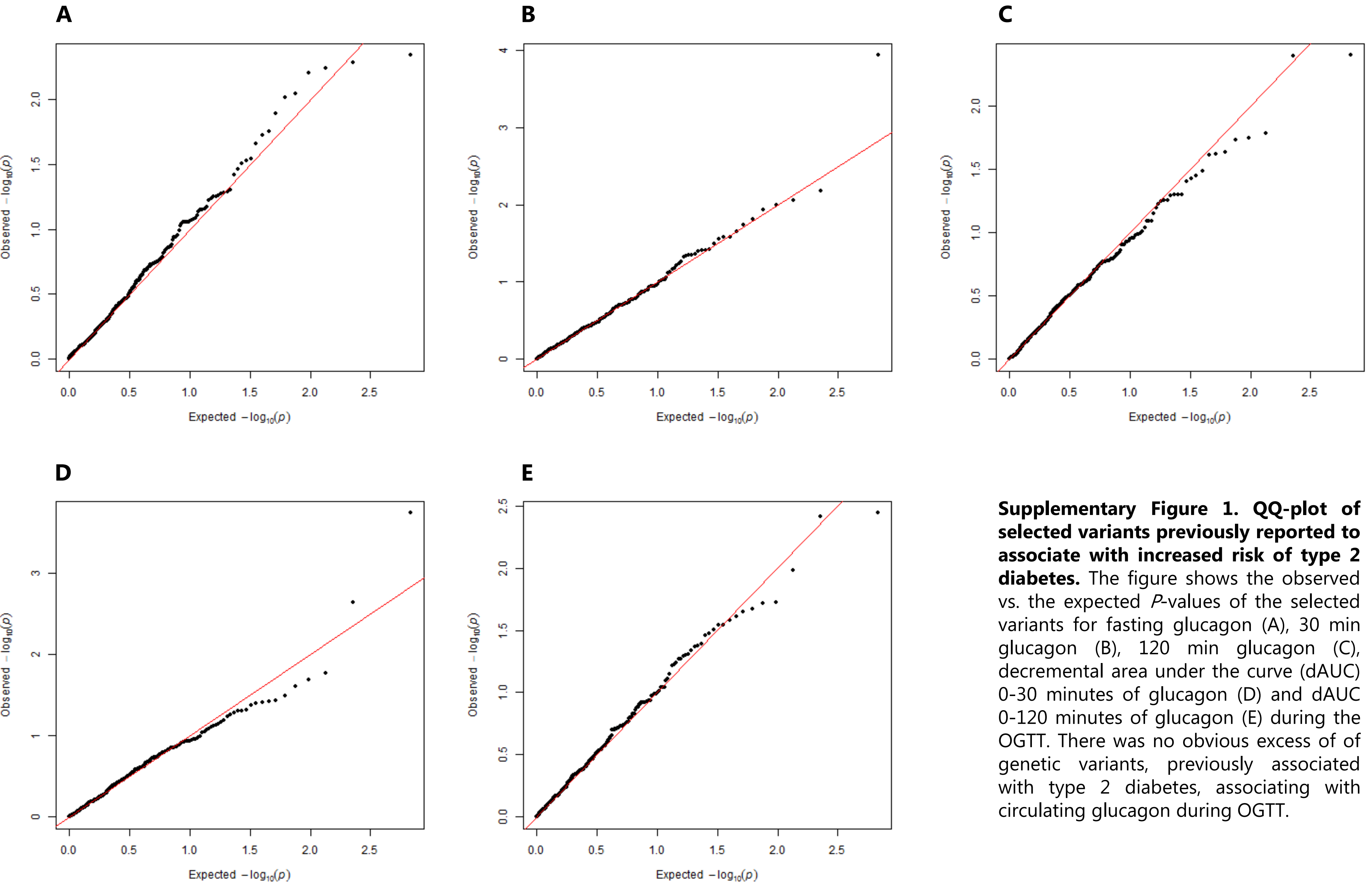

Supplementary Figure 1. QQ-plot of selected variants previously reported to associate with increased risk of type 2 diabetes. The figure shows the observed vs. the expected *P*-values of the selected variants for fasting glucagon (A), 30 min glucagon (B), 120 min glucagon (C), decremental area under the curve (dAUC) 0-30 minutes of glucagon (D) and dAUC 0-120 minutes of glucagon (E) during the OGTT. There was no obvious excess of of genetic variants, previously associated with type 2 diabetes, associating with circulating glucagon during OGTT.
